# Response of aboveground biomass and diversity to nitrogen addition along a degradation gradient in the Inner Mongolian steppe, China

**DOI:** 10.1038/srep10284

**Published:** 2015-07-21

**Authors:** Xiaotian Xu, Hongyan Liu, Zhaoliang Song, Wei Wang, Guozheng Hu, Zhaohuan Qi

**Affiliations:** 1 College of Urban and Environmental Sciences and MOE Laboratory for Earth Surface Processes, Peking University, Beijing, 100871, China; 2 School of Environment and Resources, Zhejiang Agricultural and Forestry University, Lin’an, Zhejiang 311300, China

## Abstract

Although nitrogen addition and recovery from degradation can both promote production of grassland biomass, these two factors have rarely been investigated in combination. In this study, we established a field experiment with six N-treatment (CK, 10, 20, 30, 40, 50 g N m^−2^ yr^−1^) on five fields with different degradation levels in the Inner Mongolian steppe of China from 2011–2013. Our observations showed that while the external nitrogen increased the aboveground biomass in all five grasslands, the magnitude of the effects differed with the severity of degradation. Fields with a higher level of degradation tended to have a higher saturation value (20 g N m^−2^ yr^−1^) than those with a lower degradation level ( < 10 g N m^−2^ yr^−1^). After three years of experimentation, species richness showed little change across degradation levels. Among the four functional groups of grasses, sedges, forbs and legumes, grasses shared the most similar response patterns with those of the whole community, demonstrating the predominant role that they play in the restoration of grassland under a stimulus of nitrogen addition.

In recent decades, atmospheric nitrogen deposition has become a global concern^1^; therefore, the effects of nitrogen addition on ecosystem productivity, biodiversity, and function (e.g., carbon source or sink) have been extensively studied by the scientific community^2^,^3^. Additionally, grassland degradation, as indicated by reverse succession of the plant community and coarsening of soil due to overgrazing, burrowing of small mammals, and climate change, has become a global problem^4^. About 40% of the land surface of the earth is covered by grasslands, half of which have become degraded^5^. Degraded grasslands often suffer from further reductions in species richness, ecosystem productivity and vegetation cover, causing wind erosion and nutrient loss^6^. Grassland fertilization had been used as a rapid and effective method of restoring the productivity and quality of pastures in some industrialized countries since the 1970s^7^. As a result, various fertilization experiments have been designed in different areas and across different vegetation types to identify possible effects of nitrogen addition on ecosystem biomass and species richness worldwide^8^,^9^,^10^,^11^,^12^,^13^,^14^,^15^,^16^,^17^.

Although there seems to be a universal response pattern across different grasslands to the amount of nitrogen added, such as the increase in grass growth with nitrogen addition^2^, other specific patterns may also exist. Past studies have shown that degradation status influenced the effects of nitrogen addition on biomass. A large increase of biomass was observed on a degraded grassland when only a small N-treatment was applied^18^, and a high level of added nitrogen (51 g N m^−2^ yr^−1^) has been shown to still cause significant increases in biomass, because the species of degraded grassland are often under a condition of nutrient deficiency, making demand high and imperative^19^. Conversely, on mature grassland, which could be considered as a climax ecosystem, increases in biomass in response to nitrogen are often very limited^20^. This is because community biomass cannot exceed beyond its climax state, making the system quite stable and inert to outer stimulation. The opposite result has also been observed, in that nitrogen addition led to greater increases in biomass on mature grassland than on degraded grassland due to the higher water availability of the former^11^. Thus, it is necessary to separate the influence of biomass change in response to the addition of nitrogen from that of the restoration process. However, the recovery of a disturbed ecosystem is a complex process that may contain a series of stages. Accordingly, it might be helpful to discuss variations among stages rather than simply comparing degraded and mature sites. Furthermore, it would be useful to reveal the possible benefits of nitrogen addition to the restoration process with respect to promoting growth of the community and increasing the species richness and proportion of climax species that dominate local natural grasslands.

Previous studies also revealed that the enhancing effect of nitrogen on biomass decreased with increasing nitrogen addition^2^. This phenomenon indicates that the increase in biomass caused by nitrogen addition may have a saturation value, similar to nitrogen induced eutrophication^21^. When the added amount exceeds this value, the effects on vegetation will lessen, and may even become negative^22^. Excess nitrogen cannot be absorbed by the soil and vegetation, and is therefore leached or volatilized. It might also change the physical and chemical properties of the soil, leading to a decrease in biomass^23^,^24^ and species diversity, especially richness^21^,^25^,^26^, further influencing the stability of grassland systems^27^,^28^. These effects are often explained by the favorability of high nitrogen concentration in soil to a few opportunistic species^29^,^30^,^31^, exclusion of light competitors in response to nitrogen-enhanced plant growth^32^,^33^,^34^, or exclusion of species due to soil acidification with excess nitrogen addition^35^.

As such, we investigated whether the response patterns of grassland biomass and plant species diversity to nitrogen addition change with the degradation gradient. We conducted a controlled experiment on the Ulan Buton steppe at the southeastern edge of the Inner Mongolian Plateau, China, by selecting five experimental fields for nitrogen addition, all in different stages of degradation, to examine the patterns and impacts of subsequent biomass and species change. Specifically, we attempted to answer the following questions regarding nitrogen addition: What roles do the different functional groups play in the response of plant community biomass to nitrogen addition? Would nitrogen addition benefit recovery of the degraded grasslands? Do the effects of nitrogen addition on the restoration of grassland biomass differ with degradation status?

## Results

### Effects of nitrogen addition on the total aboveground biomass

**Figure 1:**
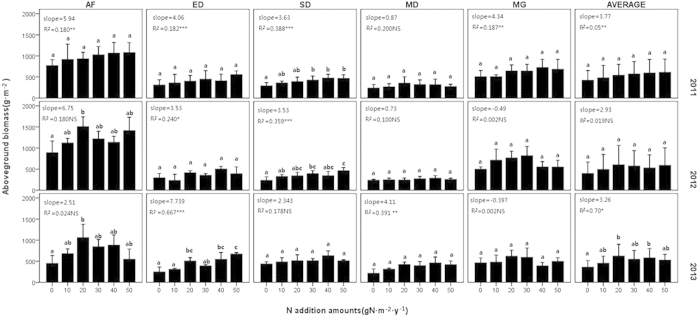
Response of total aboveground biomass to the nitrogen addition. AF = abandoned farmland, ED = extremely degraded grassland, SD = severely degraded grassland, MD = moderately degraded grassland, MG = mature grassland. Bars with the same letter (e.g., a, b) were not significantly different in tests of S-N-K. Regression parameters were estimated with N treatment in each graph. Significant differences are reported as NS, P > 0.05; *, P < 0.05; **, P < 0.01; ***, P < 0.001. The error bars indicate the standard deviation.

Biomass generally increased with increased N-treatment in all fields, as indicated by the positive slope values. Moreover, the slopes increased with the level of degradation (P = 0.007), especially in 2012. Biomass in AF (abandoned farmland, with the most severe degradation) increased most notably, with the greatest slopes being observed in the first two years. Although the general slope of AF decreased in 2013, the slope of the lower N-treatment (from CK to N-20) was still very large, reaching 30.53. In SD (severe degradation) and ED (extremely severe degradation), biomass increased with N-treatment throughout the three-year experimental period. The MD (moderate degradation) responded least significantly, with slopes remaining very close to 0 until 2013. For MG (mature grassland), the slopes were positive in 2011, but became negative in 2012 and 2013. Although the biomass of MG increased with the addition of nitrogen between N-0 and N-30 (with large slopes of 10.20 and 5.23) in 2012 and 2013, biomass decreased when nitrogen addition reached N-40 (Fig. 1).

In 2013, a saturation pattern of biomass was observed, in which treatment N-20 led to a notable increase in biomass, while saturation was reached for treatments exceeding N-20 in the five fields on average. However, no consistent pattern was observed among treatments. The saturation values for AF were N-20 in 2012 and 2013, while those for ED were N-20 in 2013 and those for SD were N-30 in 2011 and 2012. MD and MG did not show valid saturation values in any of the three years, so the possible values might be less than N-10 (Fig. 1).

### Effects of nitrogen addition on the aboveground biomass of different functional groups

**Figure 2:**
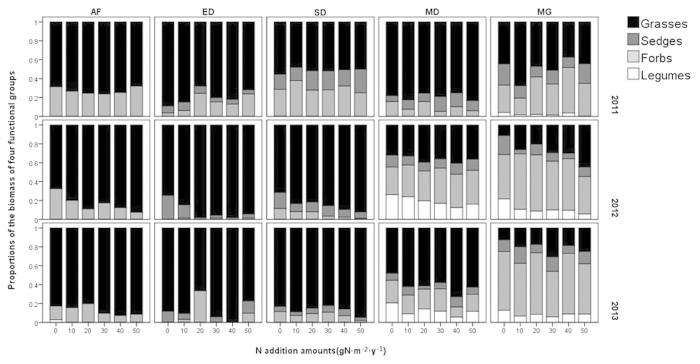
Response of the percentage of aboveground biomass of different functional groups to the nitrogen addition. AF = abandoned farmland, ED = extremely degraded grassland, SD = severely degraded grassland, MD = moderately degraded grassland, MG = mature grassland. The different colors showed in the figure represent the percentage of each functional group in the total aboveground biomass.

**Figure 3:**
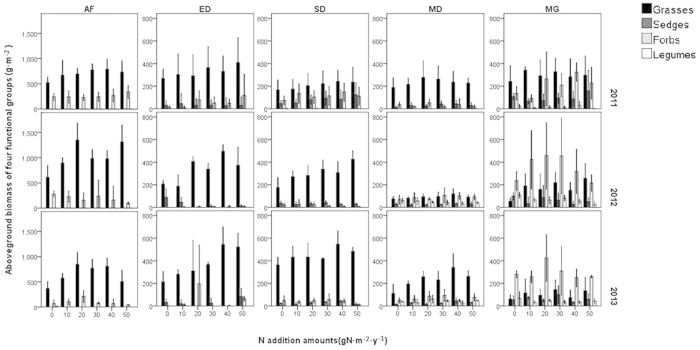
Response of aboveground biomass of different functional groups to the nitrogen addition. AF = abandoned farmland, ED = extremely degraded grassland, SD = severely degraded grassland, MD = moderately degraded grassland, MG = mature grassland. The error bars indicate the standard deviation.

The biomass of the grass group showed a significantly greater increase with nitrogen addition than that of other functional groups. Although the aboveground biomass of sedges and legumes changed during the three years (Table S1), this change had little influence on the total biomass because of their low proportion (Fig. 2). The grass group showed positive slopes, while the other groups showed negative or non-significant slopes (Table S2). The trend in the grass group was similar to that of the total biomass, which was more obvious in the degraded grassland than in the mature grassland. In AF, ED and SD, after a year of nitrogen addition the dominance of the grass group was enhanced. In the third year, this phenomenon also occurred in MD. However, MG showed the opposite trend. Nevertheless, the biomass of grass in all fields during all years increased with increasing N-treatment. In contrast, the biomass of legumes decreased significantly and the biomass of sedges and forbs showed no clear trend (Fig. 3, Table S2).

Increases in aboveground biomass of grass were significantly positively correlated with increases in total aboveground biomass, yielding a slope of 1.012 (P < 0.001). The increases in the grass group were almost the same as those of the total biomass. All five fields presented similar patterns, with MG comprising the lowest proportion of the grass group, having the greatest slopes. These results indicated that the biomass of the other functional groups (forbs, sedges and legumes) contributed little to changes in the total biomass. Moreover, increases in the total aboveground biomass in response to nitrogen addition were primarily attributed to the response of the grass group.

### Factors influencing the aboveground biomass under nitrogen addition

ANOVA in the univariate GLM showed that the degradation status and N-treatment had the greatest influence on the changes of biomass. Although some interannual variations such as temperature and precipitation may exist, the study years had little influences on biomass, implying that the natural restoration process might not influence the response of biomass to nitrogen addition within three years. We also found no difference between the two nitrogen application methods (solution or dry dust), indicating that the method of adding nitrogen did not influence the experimental results (Table S1). Since the amount of water in our solution was quite small, it was not expected to influence the soil conditions significantly.

MANOVA in the multivariate GLM showed that the N-treatment had the greatest effect on the grass group, but did not influence other groups significantly (Table S1). During the three years of the experiment, the biomass of grasses and forbs changed slightly with time, but that of sedges and especially legumes changed significantly. The sedge group decreased in biomass while legumes increased in biomass during the experimental period (Fig. 3; Table S2). All four functional groups were influenced significantly by the degradation status of the experimental fields, indicating that the condition of the fields was the most significant influencing factor.

### Effects of nitrogen addition on species richness

**Figure 4:**
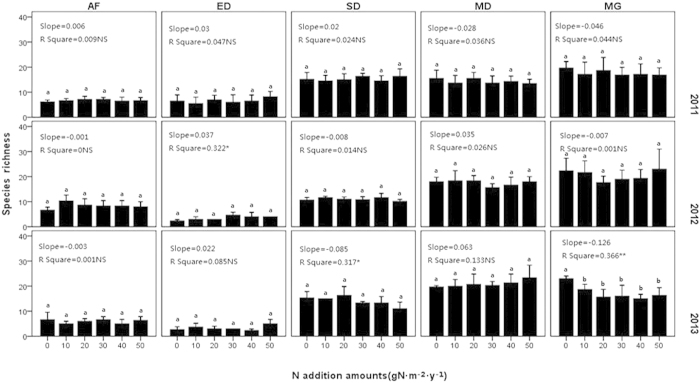
Response of species richness to the nitrogen addition. AF = abandoned farmland, ED = extremely degraded grassland, SD = severely degraded grassland, MD = moderately degraded grassland, MG = mature grassland. Bars with the same letter (e.g., a, b) were not significantly different in tests of S-N-K.

The plant species richness among the five experimental fields was quite different, but did not differ significantly among N-treatment. After three years, different trends began to appear in the slopes, although these differences were quite small. Species richness increased in the ED and MD but decreased in the MG and SD with increasing N-treatment, though changes in species richness were not significant in any sites except MG (Fig. 4).

## Discussion

The addition of nitrogen to our experimental fields generally had a positive effect on the aboveground biomass of the grasslands. Further, nitrogen saturation was observed after the three-year experiment. These results agreed well with previous studies conducted in the Inner Mongolian steppe, which had saturation values of 10.5 g N m^−2^ yr^−1^ and 12 g N m^−2^ yr^−1^ 11,12. However, this pattern differed among the five fields in our study. Although no clear trend was observed, our study nonetheless demonstrated that grasslands having undergone higher levels of degradation might have higher saturation values.

The generally positive effects of N-treatment might be explained by the relatively low soil nitrogen in the Inner Mongolian steppe, as well as the limiting effect of nitrogen on plant growth^12^. The data indicated that the response of biomass was more heavily influenced by the nitrogen addition and the degradation status of the fields than the duration of the experiment and the nitrogen addition methods. Although previous studies revealed natural restoration of biomass in degraded grasslands^36^, this effect might take a long time to appear, as indicated by our MG requiring 10 years to show the effects of natural restoration. Thus, a three-year study period is too short to explain this effect clearly, while nitrogen addition can impact the grassland much faster. Therefore, in this experiment, the addition of artificial nitrogen could be a primary factor controlling the biomass increase, with natural restoration being secondary.

The varied responses of fields with different degradation status might be associated with the local degraded conditions of the fields, such as plant species compositions and soil features. Climax species in MG could provide strong buffering capacity to biomass change, and make the community inert to nitrogen stimulus, while annuals and degraded indicators adapted to degradation would be able to use of exotic nitrogen actively and quickly, causing degraded grasslands such as AF, ED and SD to be more sensitive^37^,^38^. For the influence of soil features, the most important factor in the soil is the nitrogen content. In experimental fields with relatively high topsoil nitrogen (AF and MG), there tended to be decreasing biomass when the N-treatment reached a high level (more than N-30) (Fig. 1). These findings indicated that, in fields with high soil nitrogen content, high nitrogen enrichment could do more harm than good. Accordingly, since it had been recently abandoned from farmland, AF had poor species richness and composition, but was not lacking nutrients owing to the nitrogen accumulation during cultivation^39^,^40^. Therefore, the results of this study suggest that this field was influenced by the above two main factors as follows: a plant species composition with a high proportion of annuals and degradation indicators suggesting a high degradation status was associated with a rapid response and large slopes, while the nitrogen addition history led to a relatively high nitrogen concentration in the soil along with high biomass, resulting in a biomass decline under high N-treatment.

We also found that the biomass of the grass group played a predominant role in responding to the addition of nitrogen, which was consistent with the results of previous studies conducted in this region^15^. The lack of response of MG to N-treatment might be related to the low proportion of the grass group in this field (Fig. 2). A similar phenomenon in which nitrogen addition led to an increase of grasses and a decrease of forbs in some grasslands was also found^21^,^41^. This could be explained by the favorable effects of nitrogen on the grass group owing to their higher nitrogen use efficiency and water use efficiency than those of the forb group, as well as the subsequent growth suppression^15^,^42^. Since water resources are very limited in this semiarid area, plant species with higher water use efficiency can make better use of nutrients. Therefore, the increase of available nitrogen was of greater benefit to the grass group than other species^43^.

An obvious decrease of species richness only occurred in the MG in the third year, and none of the other fields changed significantly. This phenomenon may have been due to the relatively short experimental time. Nitrogen addition might change the biomass, height, abundance and cover faster than species richness. Our results generally agreed with those of previous studies in this region^11^,^12^ and other parts of the world^26^. Although no statistically significant differences were observed, the variations among sites with different degradation levels might be explained by light competition^32^,^33^,^34^. In ED and MD, the plant communities were always relatively open; therefore, the addition of nitrogen did not increase competition for light, but instead created a new niche with an increased nutrient supply, causing richness to increase slightly with the N-treatment. In SD, the initial community was not very dense, but its density increased from the second year. As a result, the light competition also increased with increasing nitrogen supply. MG was initially very dense and was therefore most influenced by light competition among the five fields. The species richness of SD and MG tended to decrease because of the loss of some low-growing species under increased density pressure. The results observed for MG demonstrated that the buffer effect could prevent the mature grassland from growth under exotic nutrient supply, but not the loss of species. As a result, nitrogen addition would do harm rather than aid a mature grassland.

In summary, artificial nitrogen addition was able to promote the restoration of degraded grasslands, especially their aboveground biomass, mainly by benefitting the grass group. However, nitrogen addition was not necessary for biomass accumulation on the mature steppe. Currently, most degraded grasslands are subject to grazing pressure^44^,^45^,^46^. At the beginning of this century, about 85.4% of the grassland in China was degraded, a trend that has persisted until recently^47^,^48^. In our study area of Inner Mongolia, which contains the majority of grassland in China, the area of degraded grassland has reached 22.47 million hm^2^ and is still expanding^49^. Thus, the addition of nitrogen to these degraded grasslands might favor their restoration of biomass, but facilitate transformation of the steppe into a grass-dominated community in terms of biomass, reducing the plant species evenness that characterizes the mature grassland.

## Methods

### Study area

**Figure 5:**
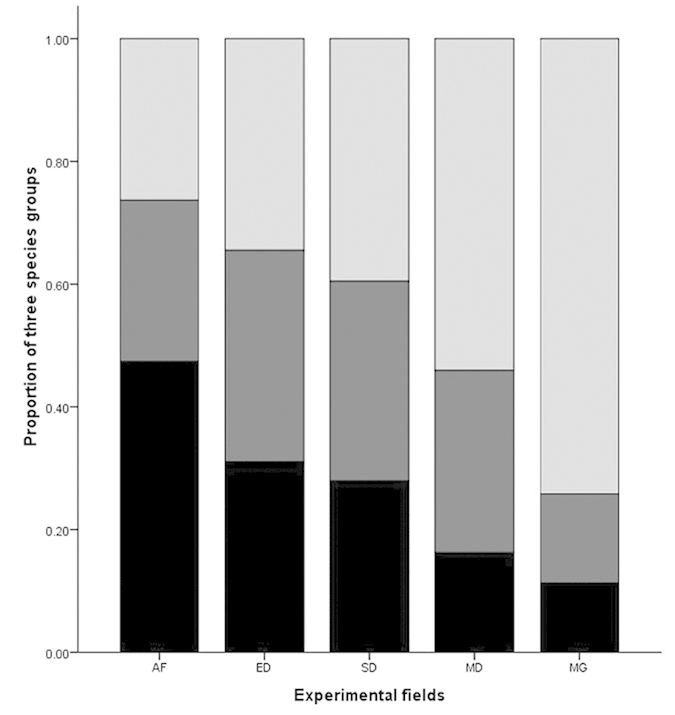
The proportion of each experimental field of three groups categorized to indicate the level of degradation in this region: pioneer species (annual, in black), intermediate species (degradation indicators, in dark grey), and climax species (in light grey). The X axis represents the degradation levels of the five fields from high to low.

Our experiment was conducted on the Ulan Buton steppe, Inner Mongolian Plateau, China. The study area is characterized by a mean annual precipitation of around 400 mm and a mean annual temperature of –1.4°C. The soil is classified as Chernozems, with sand and silt dominating its surface layer^50^. Particle size data are shown in Table 1. Five 100 m × 100 m experimental fields were fenced at the flat land surface in the spring of 2011 after communicating with local people about the history of human disturbances at each site. Among all vegetation and soil features, plant species composition and community structure can indicate the status of grassland degradation well. In previous studies, the herb species of grassland in this region could be categorized into three groups: annuals (mainly appearing in the seriously degraded steppe), moderate grazing degradation indicators, and climax species in mature steppe^50^. To quantify the grassland degradation level, we classified each site as being in one of five stages according to the proportion of the three groups of species. For example, AF had the highest proportion of annuals among the five fields and MG had the highest proportion of climax species, while the proportion of moderately grazing degradation indicators was high in the other three. The following relative covers (ranging from 0 to 1) of climax species of degraded and climax grassland, which could indicate the gradient well, were observed: abandoned farmland (AF, with 0.26), extremely degraded grassland (ED, with 0.34), severely degraded grassland (SD, with 0.40), moderately degraded grassland (MD, with 0.54), and mature grassland (MG, with 0.74) (Table 1, Fig. 5)^50^. This degradation sequence was mainly based on the meaning of the species composition degradation, but could also be adopted as a surrogate of the extent of human disturbance. At one end of the sequence is the abandoned farmland, which is heavily managed grassland at the pioneer stage of secondary succession. At the other end of the sequence is mature grassland with slight or no human disturbance.

The abandoned farmland had been sown by local inhabitants and was returned from farmland to grassland in 2010. The species composition was simple and dominated by weed annuals such as *Chenopodium acuminatum* and *Sonchus arvensis*, as well as pioneer species such as *Elymus nutans, Leymus secalinus*, and *Agropyron mongolicum* var. *villosum*.

The extremely degraded grassland, which consisted of soil covered by sand, had the lowest species richness after AF and was open to local grazing. Although climax species such as *Leymus chinensis* and *Carex korshinskyi* were present, annuals such as *Digitaria sanguinalis, Setaria viridis* and *Agriophyllum squarrosum* were dominant.

The severely degraded grassland was a high-quality pasture about two decades ago, but had degraded due to overgrazing until 2011. The flora in this area was dominated by climax species such as S*tipa baicalensis, Bromus ircutensis, Poa sphondylodes, Carex korshinskyi*, and *Potentilla longifolia*, but degradation indicator species such as *Potentilla acaulis, Artemisia frigida*, and *Artemisia capillaries* were also abundant.

The moderately degraded grassland was a pasture under management, with relatively low biomass under managed grazing. The flora was dominated by the climax species *Stipa baicalensis, Leymus chinensis, Poa sphondylodes, Potentilla longifolia*, and *Carex korshinskyi*, with much less abundant degradation indicators than observed in the severely degraded grassland.

The mature grassland was fenced from 2000 to prevent grazing and the species richness was high. The dominant species were *Leymus chinensis, Bromus inermis, Bromus ircutensis, Poa sphondylodes, Carex korshinskyi, Sanguisorba officinalis, and Bupleurum chinense*, with very few degradation indicators.

Other features of vegetation including total vegetation cover and soil features including total nitrogen content at different layers and particle size distribution all showed no clear trend along the degradation gradient (Table 1). Nevertheless, variation existed between fields. For example, MD has relatively low vegetation cover because of grazing, while AF has relatively high nitrogen content in the soil because of the history of farming and fertilization, and ED has the highest content of sand in the topsoil and therefore the lowest nitrogen content.

In our study fields, the species richness, vegetation cover and soil nitrogen content were generally lower in the degraded grasslands than in the mature grasslands (Table 1). This phenomenon was also found throughout the Inner Mongolian steppe. The reduction in vegetation cover in degraded grassland enhanced wind erosion, with fine particles blown off, resulting in the soil becoming coarser and the nitrogen poorer^50^.

### Controlled experiment design

We divided each of the fields into three blocks separated by a 2 m buffer zone. In each block, we selected 12 plots of 6 m × 6 m separated by a 1 m buffer zone for different treatments.

Nitrogen addition began in May, 2011, and the fertilizer was urine. There were six nitrogen addition amounts: 0(CK), 10, 20, 30, 40, and 50 g N m^−2^ yr^−1^.The N-treatment design was based on the response of grassland in this area and documented fertilization amounts. Nitrogen was applied four times in the first 10 days of May, June, July and August using a quarter of the annual amount at each time. Two methods of urine addition, application as a solution and dusting, were employed to examine the possible influence of the additional water on fertilizer volatility and soil moisture to assess whether the different methods used in previous studies could influence the effects of nitrogen addition. For the solution method, CK was added in the same amount of water as the other treatments, 3 L per plot per month, equal to 0.08 mm per month. This value is far less than the average precipitation, which is 50 mm in May and up to 130 mm in July.

### Sampling and measurements

The aboveground biomass was harvested in the middle of August from a 50 cm × 50 cm sampling frame. After harvest, we divided the species into four functional groups: grasses (G), sedges (S), forbs (F) and legumes (L). The biomass of each functional group was then dried in an oven at 65 °C for 48 hours. The species richness in each plot was surveyed and recorded from the end of July to the beginning of August every year.

### Data analysis

Linear regression analysis was used to calculate the trends of aboveground biomass induced by nitrogen addition in each field in each of the three years. In this study, we defined the N saturation value as when the biomass under a certain N-treatment was increased significantly relative to the CK and when the effects of the increase reached a plateau. The distinction between each N-treatment was also tested by the general linear model (GLM) post-hoc test of S-N-K.

Analysis of variance (ANOVA) based on the univariate GLM was employed to analyze the effects of factors on changes in total biomass. We used the total biomass as the dependent variable, and the experimental durations (three years), amount of added nitrogen (six treatments), degradation levels (represented by five separated fields) and nitrogen addition methods (two methods) as fixed factors. Since the nitrogen addition methods had minimal impact and the smallest significance (P = 0.796), we only considered the interactions of the other three factors. Multivariate analysis of variance (MANOVA) in the GLM was used to analyze the effects of factors on changes in biomass of different functional groups. We used the biomass of four functional groups as dependent variables, and the experimental durations (three years), nitrogen addition amounts (six treatments), and degradation levels (represented by five separated fields) as fixed factors. Their interactions were also considered.

Finally, we established a linear fit between the increases in total biomass and the increases in grass to analyze the influence of the grass group on the response to nitrogen.

The statistical software SPSS 16.0 was used to analyze all experimental data.

## Additional Information

**How to cite this article**: Xu, X. *et al*. Response of aboveground biomass and diversity to nitrogen addition along a degradation gradient in the Inner Mongolian steppe, China. *Sci. Rep*. **5**, 10284; doi: 10.1038/srep10284 (2015).

## Supplementary Material

Supplementary Information

## Figures and Tables

**Table 1:** General soil and plant community properties (means ± SD) of experimental fields.

Term	AF	ED	SD	MD	MG
Species richness	20	32	52	43	69
Vegetation cover	54%	38%	52%	29%	82%
Relative cover of climax species	0.26	0.34	0.40	0.54	0.74
Soil TN (0-20 cm, %)	0.208 ± 0.147	0.082 ± 0.015	0.190 ± 0.100	0.163 ± 0.061	0.242 ± 0.239
Soil TN (20-50 cm, %)	0.116 ± 0.029	0.123 ± 0.030	0.230 ± 0.225	0.149 ± 0.123	0.181 ± 0.031
Sand of topsoil (%)	45.7 ± 4.8	66.1 ± 3.0	57.3 ± 4.5	58.4 ± 5.6	48.8 ± 1.6
Silt of topsoil (%)	47.7 ± 5.2	28.1 ± 2.5	37.5 ± 4.2	35.8 ± 5.0	44.5 ± 1.4
Clay of topsoil (%)	6.6 ± 1.1	5.8 ± 0.5	5.1 ± 1.3	5.9 ± 0.6	6.7 ± 0.2

Note: TN = total nitrogen, AF = abandoned farmland, ED = extremely degraded grassland, SD = severely degraded grassland, MD = moderately degraded grassland, MG = mature grassland.
