# Supplementary material for: Response of aboveground biomass and diversity to nitrogen addition along a degradation gradient in the Inner Mongolian steppe, China
Source: Sci Rep. 2015 Jul 21;5:10284. doi: 10.1038/srep10284 (PMC4508527; doi:10.1038/srep10284)
Supplement: Supplementary Information [file srep10284-s1.doc]

**Response of aboveground biomass and diversity to nitrogen addition along a degradation gradient in the Inner Mongolian steppe, China**

### Xiaotian Xu^1^, Hongyan Liu^1,*^, Zhaoliang Song^2^, Wei Wang^1^, Guozheng Hu^1^, Zhaohuan Qi^1^

### * Correspondence to lhy@urban.pku.edu.cn

### 1. College of Urban and Environmental Sciences and MOE Laboratory for Earth Surface Processes, Peking University, Beijing, 100871, China

### 2. School of Environment and Resources, Zhejiang Agricultural and Forestry University, Lin’an, Zhejiang 311300, China

Table S1. Results of ANOVA in the univariate general linear model (GLM) to analyze the effects on the change of total biomass caused by the main factors; and MANOVA in the multivariate GLM to show factors affecting the change of biomass of different functional groups. The influence of year, N-treatment, degradation status and nitrogen addition method have been analyzed, and we also considered the interactions among the first three factors.

|  | Term | Functional Group | Df | F | P value |
| --- | --- | --- | --- | --- | --- |
| Test of total biomass | Year (Y) |  | 2 | 2.891 | 0.57 |
|  | **N-treatment (N)** |  | **5** | **15.916** | **<0.001** |
|  | **Degradation status (D)** |  | **4** | **209.966** | **<0.001** |
|  | Nitrogen Addition method |  | 1 | 0.67 | 0.796 |
|  | Y×N |  | 10 | 1.133 | 0.337 |
|  | **Y×D** |  | **8** | **15.158** | **<0.001** |
|  | **N×D** |  | **20** | **2.041** | **0.006** |
|  | Y×N×D |  | 40 | 1.368 | 0.077 |
| Test of four functional groups | Year (Y) | G | 2 | 2.247 | .108 |
|  |  | S | 2 | 2.674 | .071 |
|  |  | F | 2 | 4.625 | .011 |
|  |  | L | 2 | 89.279 | <0.001 |
|  | N-treatment (N) | G | 5 | 15.304 | <0.001 |
|  |  | S | 5 | .964 | .440 |
|  |  | F | 5 | 2.446 | .034 |
|  |  | L | 5 | 5.525 | <0.001 |
|  | Degradation status (D) | G | 4 | 227.654 | <0.001 |
|  |  | S | 4 | 23.035 | <0.001 |
|  |  | F | 4 | 78.295 | <0.001 |
|  |  | L | 4 | 203.803 | <0.001 |
|  | Y×N | G | 10 | 1.917 | .043 |
|  |  | S | 10 | 1.010 | .435 |
|  |  | F | 10 | 2.344 | .011 |
|  |  | L | 10 | 2.825 | .002 |
|  | Y×D | G | 8 | 21.564 | <0.001 |
|  |  | S | 8 | 2.694 | .007 |
|  |  | F | 8 | 10.020 | <0.001 |
|  |  | L | 8 | 43.089 | <0.001 |
|  | N×D | G | 20 | 2.635 | <0.001 |
|  |  | S | 20 | .815 | .695 |
|  |  | F | 20 | 1.013 | .446 |
|  |  | L | 20 | 3.607 | <0.001 |
|  | Y×N×D | G | 40 | 1.129 | .282 |
|  |  | S | 40 | .616 | .967 |
|  |  | F | 40 | .786 | .821 |
|  |  | L | 40 | 1.617 | .014 |

Note: G=grasses, S=sedges, F=forbs, L=legumes, AF=abandoned farmland, ED=extremely degraded grassland, SD=severely degraded grassland, MD=moderately degraded grassland, MG=mature grassland.

Table S2. Slopes, r squares and P values of linear regressions of effects of nitrogen addition to the biomass of four functional groups.

| Year | Field | Term | Slope | r Square | P value |
| --- | --- | --- | --- | --- | --- |
| 2011 | AF | G | 4.23 | 0.144 | 0.023 |
|  |  | S | / | / | / |
|  |  | F | 2.268 | 0.126 | 0.033 |
|  |  | L | / | / | / |
|  | ED | G | 2.455 | 0.066 | 0.129 |
|  |  | S | -0.168 | 0.002 | 0.782 |
|  |  | F | 1.775 | 0.12 | 0.038 |
|  |  | L | / | / | / |
|  | SD | G | 1.6 | 0.074 | 0.108 |
|  |  | S | 1.4 | 0.137 | 0.027 |
|  |  | F | 0.641 | 0.025 | 0.362 |
|  |  | L | -0.014 | 0.04 | 0.244 |
|  | MD | G | 0.675 | 0.016 | 0.469 |
|  |  | S | 0.436 | 0.1 | 0.06 |
|  |  | F | -0.246 | 0.021 | 0.399 |
|  |  | L | / | / | / |
|  | MG | G | 0.386 | 0.003 | 0.763 |
|  |  | S | 0.965 | 0.039 | 0.249 |
|  |  | F | 3.137 | 0.146 | 0.022 |
|  |  | L | -0.149 | 0.018 | 0.438 |
| 2012 | AF | G | 9.675 | 0.269 | 0.027 |
|  |  | S | / | / | / |
|  |  | F | -2.921 | 0.091 | 0.223 |
|  |  | L | / | / | / |
|  | ED | G | 4.84 | 0.403 | 0.005 |
|  |  | S | -1.457 | 0.318 | 0.015 |
|  |  | F | 0.147 | 0.174 | 0.085 |
|  |  | L | / | / | / |
|  | SD | G | 4.035 | 0.422 | <0.001 |
|  |  | S | -0.06 | 0.005 | 0.695 |
|  |  | F | -0.448 | 0.109 | 0.28 |
|  |  | L | / | / | / |
|  | MD | G | 0.557 | 0.127 | 0.146 |
|  |  | S | 0.114 | 0.041 | 0.42 |
|  |  | F | 0.561 | 0.078 | 0.262 |
|  |  | L | -0.5 | 0.21 | 0.056 |
|  | MG | G | 2.754 | 0.205 | 0.059 |
|  |  | S | -0.81 | 0.062 | 0.319 |
|  |  | F | -1.184 | 0.009 | 0.706 |
|  |  | L | -1.251 | 0.434 | 0.003 |
| 2013 | AF | G | 3.716 | 0.077 | 0.266 |
|  |  | S | / | / | / |
|  |  | F | -1.094 | 0.058 | 0.337 |
|  |  | L | / | / | / |
|  | ED | G | 6.821 | 0.477 | 0.002 |
|  |  | S | 0.627 | 0.062 | 0.317 |
|  |  | F | 0.291 | 0.001 | 0.884 |
|  |  | L | / | / | / |
|  | SD | G | 2.67 | 0.26 | 0.031 |
|  |  | S | 0.139 | 0.032 | 0.474 |
|  |  | F | -0.466 | 0.08 | 0.255 |
|  |  | L | / | / | / |
|  | MD | G | 3.321 | 0.377 | 0.007 |
|  |  | S | 0.423 | 0.292 | 0.021 |
|  |  | F | 0.289 | 0.022 | 0.556 |
|  |  | L | 0.076 | 0.004 | 0.804 |
|  | MG | G | 0.824 | 0.034 | 0.467 |
|  |  | S | -0.169 | 0.005 | 0.772 |
|  |  | F | -0.686 | 0.009 | 0.701 |
|  |  | L | -0.366 | 0.058 | 0.337 |

Note: G=grasses, S=sedges, F=forbs, L=legumes, AF=abandoned farmland, ED=extremely degraded grassland, SD=severely degraded grassland, MD=moderately degraded grassland, MG=mature grassland. “/” means there is no this group observed in this field.
